# Validity of Elastic Imaging Evaluation of Hamstring Muscles With Knee Contracture Using Ultrasound Shear Wave Elastography

**DOI:** 10.7759/cureus.68343

**Published:** 2024-08-31

**Authors:** Gakuto Nakao, Taiki Kodesho, Kazuma Yamagata, Kota Watanabe, Yuki Ohsaki, Masaki Katayose, Keigo Taniguchi

**Affiliations:** 1 Graduate School of Health Sciences, Sapporo Medical University, Sapporo, JPN; 2 Department of Physical Therapy, Sapporo Medical Technology, Welfare and Dentistry Professional Training College of Nishino Gakuen School Foundation, Sapporo, JPN; 3 Department of Sport Science and Research, Japan Institute of Sports Sciences (JISS), Tokyo, JPN; 4 Department of Physical Therapy, School of Health Sciences, Sapporo Medical University, Sapporo, JPN; 5 Department of Anatomy, School of Medicine, Sapporo Medical University, Sapporo, JPN

**Keywords:** mechanical properties, contracture, ultrasound elastography, elastic imaging evaluation, hamstring muscle

## Abstract

Purpose: This study used ultrasound shear wave elastography (SWE) to evaluate the mechanical properties of hamstring muscles from cadaveric specimens with knee flexion contractures.

Methods: Hamstring muscles for tensile testing were harvested from Thiel soft-embalmed cadavers with and without knee flexion contracture. Muscle specimens were mounted on a testing machine. The initial load detected when a tensile load was applied to the distal end was used as the slack length. The cross-sectional areas of the muscle at slack length were measured at the proximal and distal sites using B-mode ultrasonography. Subsequently, the muscle specimen was elongated from the slack length to 8% strain, with the shear modulus measured using SWE. Young's modulus (stress/strain) was calculated based on the displacement and tensile force obtained from the tensile test.

Results: Regression analysis showed a significant positive linear relationship between the Young's and shear moduli for all specimens at all the sites (P < 0.01 and coefficient of determination: 0.95-0.99). The Young's and average shear moduli at the proximal and distal sites were higher in all hamstring muscles with contractures than in those without contractures.

Conclusions: SWE can be used to estimate Young's moduli of hamstring muscles with contractures. Muscle specimens with contractures exhibited higher resistance to elongation, thereby indicating that their mechanical properties differed from those of muscles without contractures.

## Introduction

Contractures with joint immobility owing to cast immobilization or prolonged bed rest are caused by mechanical changes in the skeletal muscles and joint components (bone, cartilage, synovial membrane, and joint capsule), thereby limiting the range of joint motion [1]. Trudel et al. reported that, after 32 weeks of internal fixation of the rat knee joint in the flexed position, the muscles were the main cause of joint contracture up to two weeks after joint fixation [2,3]. Honda et al. created a rat model for ankle contracture, immobilized the ankle joint for 1-12 weeks, and found that the length-tension curve of the soleus muscle shifted left and upward after one week of immobility, suggesting that the skeletal muscles in the contracture model developed tension earlier in response to the changes in length than those in the control group [4]. These findings indicate that skeletal muscles with joint contractures, which are frequently subjected to physical therapy, are more resistant to elongation and have different mechanical properties compared to muscles without joint contractures. However, these methods cannot be clinically applied to humans because they involve invasive procedures [5].

Ultrasound shear wave elastography (SWE) can be a non-invasive method to evaluate the mechanical properties of tissues [6,7]. This imaging technique measures the elasticity of the tissue by assessing the speed at which shear waves, generated by sending a focused ultrasound beam to a part of the body, propagate through the tissue, which is measured by a time-of-flight algorithm in each pixel of the elastogram map in ultrafast ultrasound sequences [8]. Eby et al. reported a high correlation between the shear modulus of the swine brachialis muscle measured using this technique and Young's modulus obtained from stress-strain relationships measured in a material testing machine [9]. Furthermore, the shear modulus has been demonstrated to correlate strongly with passive force in muscles such as the adductor longus [10], rectus femoris [11], and hamstring [12] in human cadavers. Using this SWE technique, Lubin et al. investigated the effects of fixation on the mechanical properties of skeletal muscle in a rat model [13]. They found that the shear modulus of fixed skeletal muscle increased significantly by 18.1% after two weeks compared to the contralateral side. In addition, changes in mechanical properties correlated with the density of collagen from histological analysis. Because muscles with contractures have been shown to have increased collagen, which decreases their extensibility [4,14], changes in shear modulus upon elongation may differ between muscles with and without contracture. However, the application of SWE to evaluate the shear modulus of human skeletal muscles with joint contractures remains to be investigated. To this end, this study aimed to (1) investigate the relationship between the shear modulus of skeletal muscles with joint contractures obtained using SWE and their Young's modulus obtained using a material testing machine and (2) compare the mechanical properties of skeletal muscles with and without joint contractures.

## Materials and methods

Preparation of human cadavers

**Figure 1:**
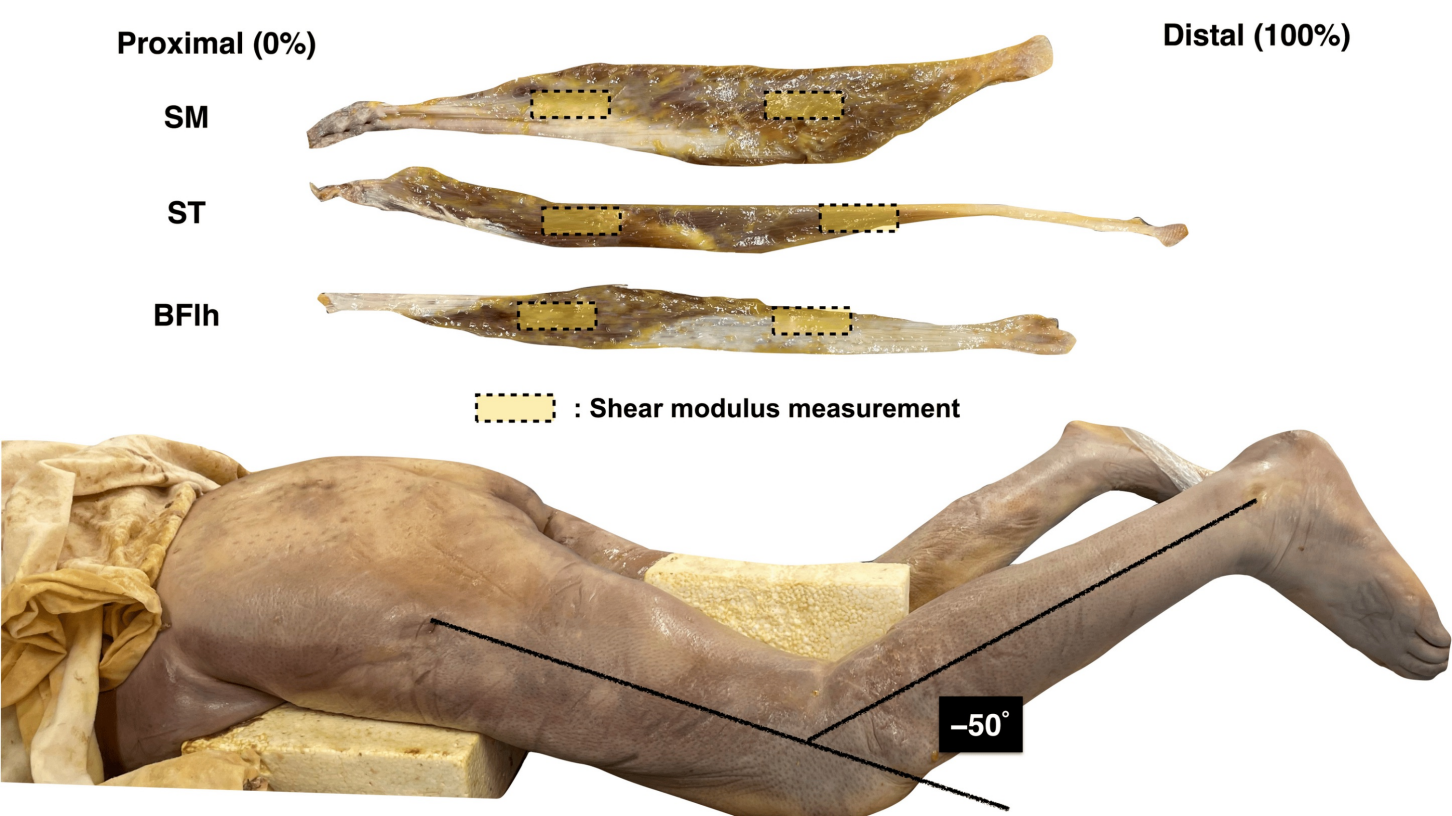
Hamstring muscles isolated from the cadaver with contracture BFlh: Biceps femoris long head, ST: Semitendinosus, SM: Semimembranosus

The subjects were Thiel soft-embalmed cadavers of females, obtained within 24 h of their death: one with knee flexion contractures (age, height, and weight at death were 96 y, 145 cm, and 50 kg, respectively) (Figure 1) and another with no injury or deformity of muscles or tendons in the hip or knee joints (age, height, and weight at death were 88 y, 145 cm, and 42 kg, respectively).

The individuals and their families had consented to body donation. The Medical Research Ethics Committee of Sapporo Medical University approved the study protocol (approval number: 4-1-70). The biceps femoris long head (BFlh), semitendinosus (ST), and semimembranosus (SM) muscles were dissected from the thigh of the cadavers and embedded using the Thiel method. Each muscle was separated from the ischial tuberosity (origin), tibia, and fibula (insertion) (Figure 1). The Thiel method is a special anatomical fixation technique that uses propylene glycol in addition to formalin [15]. Using low concentrations of formalin prevents tissue hardening [16]. Previous studies reported that the mechanical properties of muscle specimens preserved using the Thiel method are similar to those of the skeletal muscles and tendons of alive subjects [17]. Additionally, the shear modulus of the preserved specimen is similar to that of a live subject [18]. Therefore, the mechanical properties of the Thiel method-preserved hamstring muscle specimens used in our study are expected to be similar to those of skeletal muscles of live subjects. For the experiment, the muscle was immersed in a Thiel solution at a temperature of 22 °C and humidity of approximately 40% to prevent any degradation of the tissue properties of the muscle owing to changes in temperature and humidity.

Experimental protocol

**Figure 2:**
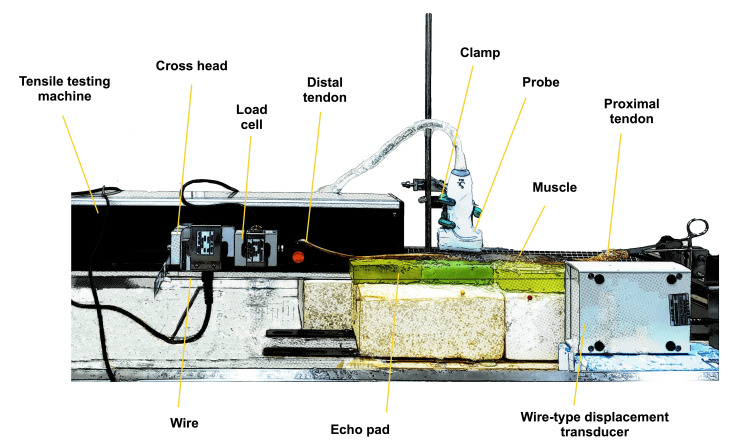
Experimental setup

The proximal and distal ends of the dissected muscle were fixed with instruments. A tabletop universal testing instrument (MCT-1150; A&D Co., Ltd., Tokyo, Japan) (Figure 2) was used to apply a tensile load to the distal end of the muscle [19].

The slack length was defined as the muscle length at the initial loading point (approximately 0.1 N). The muscle length and anatomical cross-sectional area (ACSA) at the slack length were recorded before the loading protocol was performed. The ACSA was measured at the proximal and distal sites. Based on previous research, pre-conditioning was performed over six cycles with the loading protocol used in the experiment [9]. One cycle of the loading protocol comprised applying a displacement equivalent to 8% strain from the slack length to each muscle, holding the muscle elongated for 3 s, and then returning to the slack length. After preloading, a two-cycle loading protocol was applied to each site to randomly measure the shear modulus at the proximal and distal sites of each muscle, whereas the displacement and tensile load were measured simultaneously. These measurements were continuously obtained at the proximal and distal sites from 0% to 8% strain, respectively. The test speed was set to an average of 0.90 L0/s (ranging from 0.75 to 0.97% L0/s, average slack length 374.3 mm in the 345-443 mm range), considering that the sampling frequency of the elastic images was 2.0 Hz (0.50 s period).

Measurement of muscle architecture

A measuring tape was used to measure the muscle length in increments of 0.1 cm between the proximal and distal ends of the separated tendons. The ACSA of the muscle was measured at two sites-the proximal (33%) and distal (67%), by trisecting a line connecting the proximal (0%) and distal (100%) ends of the long axis of the muscle at slack length using B-mode ultrasonography (Aixplorer Ver. 12, MSK mode; Hologic, Marlborough, MA, USA).

Measurement of displacement and tensile force

The displacement and tensile load of specimens were measured using the tensile tester (MCT-1150; A&D Co., Ltd., Tokyo, Japan), wire-type displacement transducer (special order, Assist Inc., Tokyo, Japan), load cell (LC1205-K050, A&D Co., Ltd., Tokyo, Japan), and digital indicator (TD-700T, TEAC Co., Tokyo, Japan) (Figure 2). The load cell was attached to the end of the crosshead and connected to a digital indicator to measure the tensile load. The wire in the displacement transducer was fixed to a load cell, and the displacement was measured simultaneously with the tensile load. The electrical signals of the tensile load and displacement were recorded using an analog-to-digital converter (Power Lab ML880, AD Instruments Co., Ltd., Bella Vista, NSW, Australia) and stored using Chart software (Lab Chart 8, Ver 8.1.17, AD Instruments Co., Ltd., Bella Vista, NSW, Australia). The sampling frequency was set to 1 kHz.

Measurement of shear modulus

The shear modulus of the specimen was measured at the proximal (33%) and distal (67%) sites of the total muscle length connecting the proximal (0%) and distal (100%) ends using SWE (Aixplorer Ver. 12, MSK mode; Hologic) (Figure 1), which was equipped with a linear ultrasound transducer (50 mm, 18-5 MHz). The transducer was placed parallel to the long axis of the muscle and clamped to ensure a constant transducer position and probe tilt angle during the loading protocol. A 30-mm-thick ultrasound echo pad (Echo PAD; Yasojima Proceed Co., Ltd., Amagasaki, Japan) was placed under the muscle. Ultrasound gel was applied between the echo pad and muscle and between the echo pad/muscle and transducer/muscle. The elastic color map was placed in a rectangular area (dimensions of 25 and 20 mm) that included several clear fascicles without any obvious connective tissue. Longitudinal muscles and elastic color images were recorded as movies using the Epiphan capture tool (Ver. 3.30.2.10, Argo Co.). The movies were synchronized with other digital data using Epiphan video (DVI2USB 3.0, Argo Co.) and Lab Chart 8 (Ver 8.1.17, AD Instruments Co., Ltd.) and recorded on a personal computer at 1.8 Hz. In Aixplorer, the elastic color image changes at 1.8 Hz intervals. Hence, the video was also recorded at 1.8 Hz.

The SWE generates a shear wave within soft tissues. Based on the velocity of shear wave propagation c, Young’s modulus E was quantified in kPa. The value of E was mapped in the region of interest (ROI) using color. From each pixel of the ROI, the value of E was calculated using E = 3ρc3, where density ρ was assumed to be constant (1,000 kg/m^3^) for the human soft tissue [6]. The employed device calculates E assuming that the biological tissue is isotropic, whereas the muscle is anisotropic (i.e., its mechanical properties are not identical in all directions) [6]. Accordingly, we analyzed the shear modulus by dividing the obtained E value by 3 [20].

Data analysis

The images of the cross-sectional areas were analyzed using ImageJ v1.53k (National Institutes of Health, USA). The number of pixels on a 1 cm scale on a B-mode image was measured. This measurement was set as the 1-cm pixel number for ImageJ analysis. High-echo images representing the epimysium were selected using polygon selection, and the area inside the selected region was taken as the ACSA. The average of two ACSA measurements at each site was used to calculate the stress.

**Figure 3:**
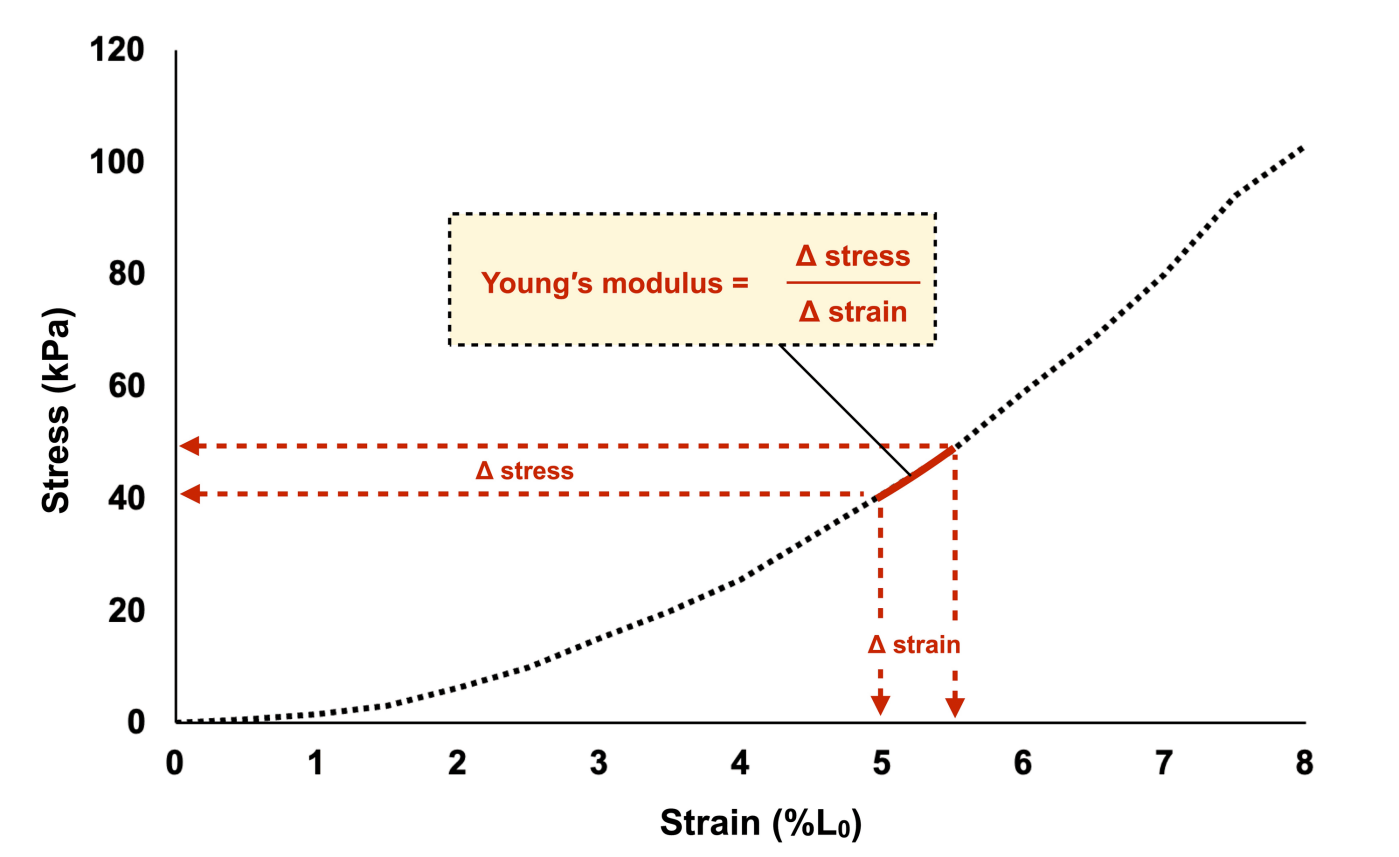
Representative examples of stress-strain curves (obtained using a tensile testing machine) for the biceps femoris long head from a cadaver with contractures and calculation of Young's modulus

Strain and stress were calculated by extracting the displacement and tensile load corresponding to a range of 0-8% strain in 1-s increments from the data recorded by the video capture module (Lab Chart 8) (Figure 3).

We obtained the strain by dividing the amount of change in original length by the original length and plotted the stress-strain curve using ACSA calculated from the initial measurements. We defined Young′s modulus as the slope between seventeen consecutive data points from the stress-strain curve, regarding the previous studies [19]. Using this approach, Young′s modulus was calculated for each of the seventeen SWE time points.

The SWE data were exported as elasticity images in the JPEG format and analyzed using custom analysis software (S-17115 Ver.1.3.0; Takei Scientific Instrument Co., Ltd., Niigata, Japan). To determine the images to be analyzed, two elastic images were extracted at 1-s increments from the image data temporally synchronized with the displacement and tensile load values in the recorded video capture module (Lab Chart 8). A rectangular ROI (20 and 5 mm wide and high, respectively) was placed on the color map. The average of two shear modulus measurements for each condition was used for statistical analysis.

All statistical analyses were performed using IBM SPSS Statistics for Windows, Version 28 (Released 2021; IBM Corp., Armonk, New York, United States). To examine the relationship between Young's modulus calculated using the material testing machine and the shear modulus measured using SWE, the Young's modulus-shear modulus relationships of the muscle with and without contracture were analyzed using linear regression.

## Results

**Figure 4:**
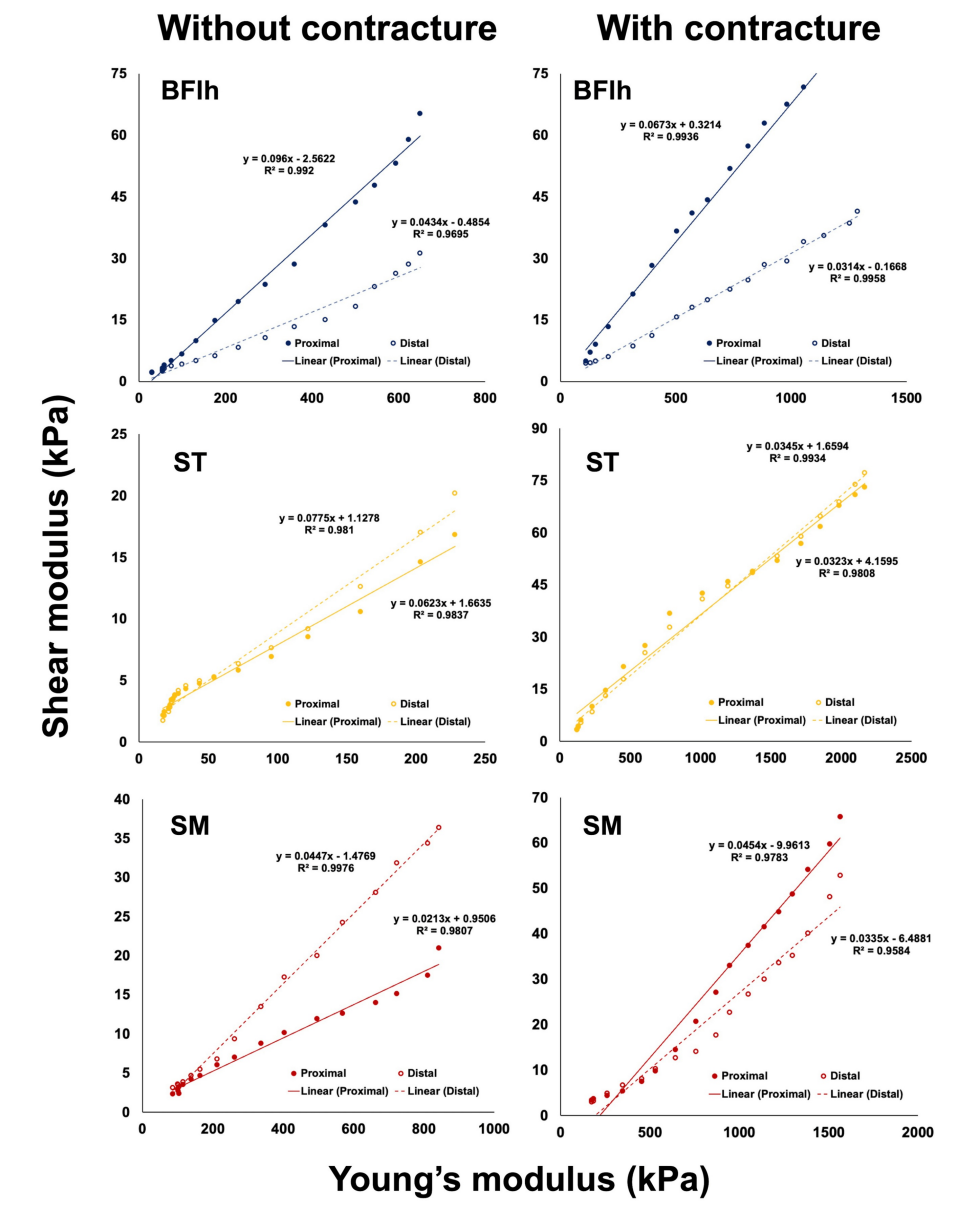
Relationship between shear and Young's moduli of cadaver hamstring muscles with and without contraction BFlh: Biceps femoris long head, ST: Semitendinosus, SM: Semimembranosus

The Young's and shear moduli for all hamstring muscles at each site were linearly correlated (P < 0.01, coefficient of determination R^2^: 0.95-0.99) (Figure 4).

**Figure 5:**
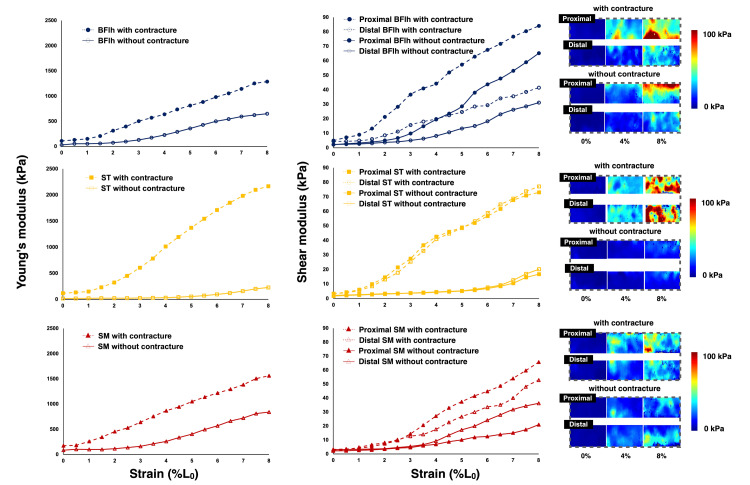
Changes in Young's modulus (left) and site-specific changes in shear modulus of each hamstring muscle (right) during tensile tests BFlh: Biceps femoris long head, ST: Semitendinosus, SM: Semimembranosus

The Young's modulus of all the muscles with contractures calculated using the material testing machine at 8% strain, wherein the most elongation was applied, was higher than that of the muscles without contracture (BFlh with and without contracture: 1286.9 and 649.6 kPa, respectively; ST with and without contracture: 2167.2 and 228.1 kPa, respectively; SM with and without contracture: 1563.5 and 842.4 kPa, respectively) (Figure 5, left). The mean shear moduli of the proximal and distal sites in all the muscles with contractures were higher than the corresponding values for the muscles without contractures, similar to Young's modulus results (BFlh with and without contracture: 62.8 and 48.2 kPa, respectively; ST with and without contracture: 75.1 and 18.5 kPa, respectively; SM with and without contracture: 59.3 and 28.7 kPa, respectively) (Figure 5, right). The shear modulus for each region of the muscle with contracture was similar to that of ST (73.1 kPa proximally and 77.1 kPa distally), whereas those of BFlh (84.1 kPa proximally and 41.5 kPa distally) and SM (65.7 kPa proximally and 52.9 kPa distally) showed higher values for the proximal region (Figure 5, right). In ST and SM, the shear modulus of the proximal and distal sites of the muscle with contracture was also higher than that of the muscle without contracture, but in BFlh, the shear modulus was higher at the proximal site of the muscle without contracture than at the distal site of the muscle with contracture (Figure 5, right).

## Discussion

In this study, the BFlh, ST, and SM were harvested from cadavers with and without contractures. The Young's and shear moduli at the proximal and distal sites of the specimens were measured based on muscle elongation. We found a strong positive correlation between the Young's and shear moduli in all muscles in all regions. The relationship between the shear modulus and passive muscle force has been demonstrated in animal [21] and human [10-12] muscle tissues. However, the relationship between the shear and Young's moduli has only been reported in one study [9] on the swine brachialis muscle. Hence, this is the first study to use SWE to investigate the mechanical properties of hamstring muscles with knee contracture.

Young's modulus is calculated from the stress and strain generated when a tensile load is applied. The higher the Young's modulus, the greater the stress required to restore the generated strain, indicating that the material is less susceptible to deformation [9]. The relationship between the shear modulus and passive muscle force in previous studies was based on the change in shear modulus in response to tensile load. However, stress was not evaluated [10-12,21]. In this study, the muscle elongation per unit area was evaluated by calculating Young's modulus (obtained from the relationship between the strain, which is the amount of elongation per unit length) and stress (which is the tensile load divided by the cross-sectional area of the tissue). A strong positive linear relationship between the Young's and shear moduli was observed for all the muscles and sites examined, with R^2^ > 0.95. This result indicates that the shear modulus measured using SWE may explain more than 95% of Young's modulus in the human muscle.

The Young's moduli calculated in this study were higher in muscles with contractures than in those without contractures. These results suggest that muscles with contractures are more resistant to passive elongation and may have mechanical properties different from those without contractures. These results correspond with those of Honda et al., who reported that the length-tension curve of the rat soleus muscle deviated to the left and upward after a period of immobility, suggesting that joint contractures associated with immobility increased Young's modulus of the skeletal muscle during muscle elongation [4]. One explanation for the increase in Young's modulus of the skeletal muscle during muscle elongation associated with immobility is fibrosis, which is associated with collagen hyperplasia within muscle fibers. Honda et al. conducted histological and biochemical studies of skeletal muscles and reported that, in muscles with joint contracture, thickening was observed in the perimysium and endomysium and the collagen content was high, which may increase the mechanical stress in the skeletal muscle [4]. However, this study could not determine the factors that caused the high Young's modulus in the muscle with contractures or whether fibrosis was generated or had progressed in the muscle. Further histological and biochemical studies are required to investigate the factors involved in contracture pathogenesis.

In this study, different values between the sites were obtained only for the shear wave velocities of the BFlh and SM. Previous studies have suggested that this intramuscular heterogeneity between sites is related to differences in the morphological characteristics of skeletal muscles, such as fascicle length and cross-sectional area [22]; however, there is no clear evidence to prove this. Our study showed that the shear moduli of muscles with contractures may not be uniform across sites. In recent years, various studies have reported on the usefulness of SWE for evaluating skeletal muscle stiffness in patients with various diseases, such as those of the rectus femoris and vastus lateralis muscles in patients with Osgood-Schlatter disease [23] and the semitendinosus muscle in patients with cerebral palsy [24], whose shear modulus was higher than those of the healthy group. However, all these values were measured at only one point. Although previous studies using SWE on healthy subjects have already shown that shear modulus in the muscle is not uniform across sites [25], we showed that shear moduli were different for proximal and distal sites in the extended position for skeletal muscles with joint contractures and skeletal muscles without joint contractures. The high correlation of the shear modulus-Young's modulus relationship for both proximal and distal sites suggests that the mechanical properties of muscles with contractures can be evaluated using SWE at multiple sites.

The limitations of this study are as follows: First, the sample size was limited to only two cadavers, one with contractures and one without. The small sample size may affect the generalizability of the findings, and future studies with larger sample sizes are necessary to confirm these results. Additionally, both cadavers were female and of advanced age (96 and 88 years, respectively). In a previous study in which muscles were sampled at the muscle bundle level in young and elderly subjects and resistance to mechanical pulling was examined, it was found that the older subjects had significantly higher tensile tension [26]. Although this was a comparison between younger and older subjects, it suggests that age may have influenced the results of the study in which tensile testing was performed. The results of this study would benefit from further validation through comparisons with cadavers across a broader range of ages, not just those closer in age. Second, while this study aimed to investigate the causes of joint contractures, it was unable to identify the specific mechanisms responsible for the observed contractures. As demonstrated in previous studies [13], muscles associated with joint contractures exhibit altered mechanical properties, a finding that was also confirmed in this study. This suggests that the underlying cause of a range of motion restrictions may involve the muscles themselves. However, the precise contribution of various tissues to joint contractures remains unclear. Further research is needed to clarify the underlying mechanisms of contracture development, particularly the roles of various joint structures. Third, the study assumes a constant soft tissue density of 1,000 kg/m³ for the calculation of Young's modulus, as reported in previous literature. Although this value is widely accepted, the variation in the density of different soft tissues could potentially influence the accuracy of the modulus measurements. For example, Faucitano et al. demonstrated that fat content within individual muscle fascicles can vary throughout the same muscle [27]. Muscles with a higher fat content would logically have a lower density compared to leaner muscles, given that fat has a significantly lower density of approximately 0.936 g/cm³. Further studies should rigorously consider these density variations to enhance the precision of modulus measurements across different muscle types and anatomical regions. Lastly, the shear modulus measured in this study represents only a local region of the muscle and may not reflect the overall mechanical properties of the whole muscle. While SWE provides valuable insights into the mechanical properties of muscle tissue, it is essential to acknowledge that the measurements obtained may not be entirely representative of the muscle as a whole. Furthermore, we examined the relationship between local shear modulus and Young's modulus for the skeletal muscle but did not distinguish the material properties of other tissues that make up the muscle-tendon complex, such as tendons. The values obtained do not reflect Young's modulus of the local skeletal muscle, because the material properties of the tissues differ among the components of the skeletal muscle [28]. Future studies should aim to assess the whole muscle's elasticity while considering these factors to provide a more comprehensive understanding of muscle mechanical properties.

## Conclusions

In this study, a significant positive linear relationship between the Young's and shear moduli was found in the proximal and distal sites of each hamstring muscle, independent of the presence of joint contractures. This result indicates that SWE may be useful for estimating Young's modulus, a measure of muscle stiffness. Additionally, the Young's and mean shear moduli at the proximal and distal sites were higher in all hamstring muscles with contractures than in those without contractures. These results suggest that muscles with contractures are more resistant to passive elongation and may have mechanical properties different from those without joint contractures.

## References

[1] Wang F, Zhang QB, Zhou Y, Chen S, Huang PP, Liu Y, Xu YH (2019). The mechanisms and treatments of muscular pathological changes in immobilization-induced joint contracture: a literature review. Chin J Traumatol.

[2] Trudel G, Uhthoff HK (2000). Contractures secondary to immobility: is the restriction articular or muscular? An experimental longitudinal study in the rat knee. Arch Phys Med Rehabil.

[3] Trudel G, Zhou J, Uhthoff HK, Laneuville O (2008). Four weeks of mobility after 8 weeks of immobility fails to restore normal motion: a preliminary study. Clin Orthop Relat Res.

[4] Honda Y, Tanaka M, Tanaka N (2018). Relationship between extensibility and collagen expression in immobilized rat skeletal muscle. Muscle Nerve.

[5] Binder-Markey BI, Sychowski D, Lieber RL (2021). Systematic review of skeletal muscle passive mechanics experimental methodology. J Biomech.

[6] Hug F, Tucker K, Gennisson JL, Tanter M, Nordez A (2015). Elastography for muscle biomechanics: toward the estimation of individual muscle force. Exerc Sport Sci Rev.

[7] Eby SF, Cloud BA, Brandenburg JE (2015). Shear wave elastography of passive skeletal muscle stiffness: influences of sex and age throughout adulthood. Clin Biomech (Bristol, Avon).

[8] Pimenta R, Coelho F, Correia JP, Vaz JR (2024). Influence of transducer pressure and examiner experience on muscle active shear modulus measured by shear wave elastography. Radiography (Lond).

[9] Eby SF, Song P, Chen S, Chen Q, Greenleaf JF, An KN (2013). Validation of shear wave elastography in skeletal muscle. J Biomech.

[10] Kato T, Taniguchi K, Kodesho T, Nakao G, Yokoyama Y, Saito Y, Katayose M (2022). Adductor longus: an anatomical study to better understand groin pain. Clin Anat.

[11] Kodesho T, Taniguchi K, Kato T (2021). Relationship between shear elastic modulus and passive force of the human rectus femoris at multiple sites: a Thiel soft-embalmed cadaver study. J Med Ultrason (2001).

[12] Nakao G, Kodesho T, Kato T (2023). Relationship between shear elastic modulus and passive muscle force in human hamstring muscles using a Thiel soft-embalmed cadaver. J Med Ultrason (2001).

[13] Lubin P, Zidi M (2024). Mechanical properties change of immobilized skeletal muscle in short position measured by shear wave elastography and pure shearing test. J Mech Behav Biomed Mater.

[14] Suwankanit K, Shimizu M (2022). Rat model of quadriceps contracture by joint immobilization. Biology.

[15] Thiel W (1992). The preservation of the whole corpse with natural color (Article in German). Ann Anat.

[16] Benkhadra M, Bouchot A, Gérard J (2011). Flexibility of Thiel's embalmed cadavers: the explanation is probably in the muscles. Surg Radiol Anat.

[17] Hohmann E, Keough N, Glatt V, Tetsworth K, Putz R, Imhoff A (2019). The mechanical properties of fresh versus fresh/frozen and preserved (Thiel and Formalin) long head of biceps tendons: a cadaveric investigation. Ann Anat.

[18] Joy J, McLeod G, Lee N, Munirama S, Corner G, Eisma R, Cochran S (2015). Quantitative assessment of Thiel soft-embalmed human cadavers using shear wave elastography. Ann Anat.

[19] Nakao G, Kodesho T, Yamagata K, Watanabe K, Ohsaki Y, Katayose M, Taniguchi K (2024). Stress-strain relationship of individual hamstring muscles: a human cadaver study. J Mech Behav Biomed Mater.

[20] Royer D, Gennisson JL, Deffieux T, Tanter M (2011). On the elasticity of transverse isotropic soft tissues (L). J Acoust Soc Am.

[21] Koo TK, Guo JY, Cohen JH, Parker KJ (2013). Relationship between shear elastic modulus and passive muscle force: an ex-vivo study. J Biomech.

[22] Kellis E, Galanis N, Natsis K, Kapetanos G (2010). Muscle architecture variations along the human semitendinosus and biceps femoris (long head) length. J Electromyogr Kinesiol.

[23] Enomoto S, Oda T, Sugisaki N, Toeda M, Kurokawa S, Kaga M (2021). Muscle stiffness of the rectus femoris and vastus lateralis in children with Osgood-Schlatter disease. Knee.

[24] Smith LR, Lee KS, Ward SR, Chambers HG, Lieber RL (2011). Hamstring contractures in children with spastic cerebral palsy result from a stiffer extracellular matrix and increased in vivo sarcomere length. J Physiol.

[25] Miyamoto N, Kimura N, Hirata K (2020). Non-uniform distribution of passive muscle stiffness within hamstring. Scand J Med Sci Sports.

[26] Pavan P, Monti E, Bondí M (2020). Alterations of extracellular matrix mechanical properties contribute to age-related functional impairment of human skeletal muscles. Int J Mol Sci.

[27] Faucitano L, Rivest J, Daigle JP, Gariepy C (2004). Distribution of intramuscular fat content and marbling within the longissimus muscle of pigs. Canadian J Animal Sci.

[28] Ward SR, Winters TM, O'Connor SM, Lieber RL (2020). Non-linear scaling of passive mechanical properties in fibers, bundles, fascicles and whole rabbit muscles. Front Physiol.

